# Evolutionary History and Novel Biotic Interactions Determine Plant Responses to Elevated CO_2_ and Nitrogen Fertilization

**DOI:** 10.1371/journal.pone.0114596

**Published:** 2014-12-05

**Authors:** Rachel Wooliver, John K. Senior, Jennifer A. Schweitzer, Julianne M. O'Reilly-Wapstra, J. Adam Langley, Samantha K. Chapman, Joseph K. Bailey

**Affiliations:** 1 Department of Ecology and Evolutionary Biology, University of Tennessee, Knoxville, Tennessee, United States of America; 2 School of Biological Sciences, University of Tasmania, Hobart, TAS, Australia; 3 National Centre for Future Forest Industries, University of Tasmania, Hobart, TAS, Australia; 4 Department of Biology, Villanova University, Villanova, Pennsylvania, United States of America; Tennessee State University, United States of America

## Abstract

A major frontier in global change research is predicting how multiple agents of global change will alter plant productivity, a critical component of the carbon cycle. Recent research has shown that plant responses to climate change are phylogenetically conserved such that species within some lineages are more productive than those within other lineages in changing environments. However, it remains unclear how phylogenetic patterns in plant responses to changing abiotic conditions may be altered by another agent of global change, the introduction of non-native species. Using a system of 28 native Tasmanian *Eucalyptus* species belonging to two subgenera, *Symphyomyrtus* and *Eucalyptus*, we hypothesized that productivity responses to abiotic agents of global change (elevated CO_2_ and increased soil N) are unique to lineages, but that novel interactions with a non-native species mediate these responses. We tested this hypothesis by examining productivity of 1) native species monocultures and 2) mixtures of native species with an introduced hardwood plantation species, *Eucalyptus nitens*, to experimentally manipulated soil N and atmospheric CO_2_. Consistent with past research, we found that N limits productivity overall, especially in elevated CO_2_ conditions. However, monocultures of species within the *Symphyomyrtus* subgenus showed the strongest response to N (gained 127% more total biomass) in elevated CO_2_ conditions, whereas those within the *Eucalyptus* subgenus did not respond to N. Root:shoot ratio (an indicator of resource use) was on average greater in species pairs containing *Symphyomyrtus* species, suggesting that functional traits important for resource uptake are phylogenetically conserved and explaining the phylogenetic pattern in plant response to changing environmental conditions. Yet, native species mixtures with *E. nitens* exhibited responses to CO_2_ and N that differed from those of monocultures, supporting our hypothesis and highlighting that both plant evolutionary history and introduced species will shape community productivity in a changing world.

## Introduction

Several analyses of primary productivity at community, biome and global levels have indicated that soil nitrogen (N) generally limits carbon sequestration, but have failed to address whether individual species respond differently to increased soil N [1]–[4]. In contrast to the implied paradigm that all plants should produce more biomass in response to increased soil N, a growing body of research shows that not all species respond positively, or even at all, to increased soil N, especially in elevated CO_2_ conditions [5]–[10]. This is likely because plants have evolved different capacities to compete for soil resources [11]. For example, a species whose traits reflect an evolved ability to strongly compete for soil N would accumulate more biomass in response to increases in soil N, thereafter increasing in dominance over species whose traits reflect an evolved tradeoff to compete along some other niche axis [8]. Ultimately, anthropogenically increased levels of atmospheric CO_2_ and soil N will alter species abundance, composition and diversity, which will in turn impact many important ecosystem processes and functions [7], [12], [13]. Understanding how past evolution and contemporary biotic interactions shape plant species responses to environmental change could provide key insight into how plant diversity and function may be altered in global change scenarios.

Phylogenetic information can be used to explain patterns in species responses to global change, which in turn can explain the past evolution of species niche spaces [14], [15]. Several studies have shown that more phylogenetically diverse plant communities produce more biomass on average, indicating that species niche spaces (and, by association, traits for resource acquisition and processing) are more similar among species with more shared evolutionary history than those with less shared evolutionary history [16]–[18]. These studies provide strong evidence that niches of closely related species are phylogenetically preserved (i.e., exhibit phylogenetic niche conservatism [PNC]), reflecting ancestral traits that through various evolutionary processes [15], [19] have changed little through evolutionary time [20], [21]. Empirical evidence confirms that leaf and root traits, which are important indicators of resource uptake capacity and competitive ability, are phylogenetically conserved within, and are unique to, plant lineages [22], [23]. If this pattern is consistent across plant lineages, we should expect that more closely related plant species would respond more similarly to environmental change. In support of this prediction, Davis et al. (2010) have found that species within certain angiosperm lineages flower earlier in the year in response to a warming climate and may thus be more favored than other lineages as global temperatures rise [24]. Although PNC is certainly not universal [21], it may be strong enough in specific plant traits and lineages such that evolutionary history alone can sufficiently explain patterns in plant species productivity, especially in the context of global change [14], [15].

Despite evidence that evolutionary history can be used to predict patterns of plant responses to globally altered abiotic conditions [14], [24], it remains unclear how community responses to environmental change may be altered by introductions of non-native species [25]. A warming climate and altered soil nutrient levels will have variable effects on the productivity of plant species depending on the axes along which respective species are specialized [5], [11], as well as open niche spaces that can be filled by non-native species [26]. In particular, increased soil N can facilitate the establishment and proliferation of fast growing, nitrophilic non-natives at the expense of slow-growing natives whose capacities for N uptake and processing are more limited (i.e., whose growth strategies are more conservative) [7], [27]. Once established in a new range, non-native species can create plant-soil feedbacks that increase [28] or decrease [29] native species productivity. The consequences of these feedbacks have been shown to be greater in situations where native and introduced species share less evolutionary history (i.e., the interaction is more ecologically ‘novel’) [30], [31]. Thus, introduced species should have different influences on communities with different evolutionary backgrounds. Amidst increasing rates of non-native species across the globe [32], understanding the interacting effects of abiotic changes and species introductions on native plant productivity represents a critical challenge to address in global change research.

To determine how evolutionary history and novel biotic interactions shape plant responses to abiotic agents of global change, we analyzed biomass production of eucalypt species monocultures and mixtures with an introduced species across elevated CO_2_ and increased soil N conditions. Specifically, we used 28 *Eucalyptus* species in two subgenera, *Eucalyptus* and *Symphyomyrtus*, that are native to the island state of Tasmania, Australia, where they co-dominate sub-alpine to coastal forest and shrubland ecosystems [33]. Despite their ecological and economic prominence in Australia, the magnitude and direction in which agents of global change alter the ecology of these species has scarcely been investigated (but see [34]), thus representing an ideal system for better understanding plant responses to global change. Using subgenus as an indicator of evolutionary history, we hypothesized that more closely related species would share more similar productivity responses to experimentally manipulated atmospheric CO_2_ and soil N, as research shows that species with greater shared evolutionary history have more similar resource acquisition traits [22], [23], but that novel biotic interactions would alter evolutionarily-based plant productivity responses to changing abiotic conditions. Subgeneric differences in productivity responses to abiotic global change factors would suggest that species within the same lineage have inherited similar traits for resource use (i.e., support for PNC), which should be confirmed by subgeneric differences in resource-use traits (e.g., ratio of root to shoot biomass). Further, effects of species interactions on responses to abiotic changes would indicate that biotic agents of global change can also mediate patterns of plant productivity.

## Methods

We used a design whereby two individuals were planted per pot, and pairs were composed of two same-species native individuals or one native individual with one introduced, non-native individual of *Eucalyptus nitens*. Plants were grown in a greenhouse setting (alternating high and low CO_2_ conditions weekly) and treated with factorial combinations of elevated vs. ambient atmospheric CO_2_ and high vs. low soil N. After six months of growth, plants were harvested for biomass. We analyzed patterns in total, aboveground and belowground biomass production, as well as the ratio of root to shoot biomass, as these reflect the evolutionary basis of resource acquisition strategy and ecosystem functioning.

### Focal species

To determine independent and interactive effects of three global change factors (CO_2_, N and novel species interaction) and phylogenetic relatedness on plant productivity, we used 28 of the 30 species within the genus *Eucalyptus* (family Myrtaceae) that are native to Tasmania. Native individuals were paired with either a conspecific or the tree species *E. nitens*, which is non-native to Tasmania, and grown in varying atmospheric CO_2_ and soil N concentrations. The native species have been phylogenetically and morphologically separated into two subgenera: *Eucalyptus* (*E. amygdalina, E. coccifera, E. delegatensis, E. nitidia, E. obliqua, E. pauciflora, E pulchella, E. radiata, E. regnans, E. risdonii*, *E. sieberi*, and *E. tenuiramis*) and *Symphyomyrtus* (*E. barberi, E. brookeriana, E. cordata, E. dalrympleana, E. globulus, E. gunii, E. johnstonii, E. morrisbyi, E. ovata, E. perriniana, E. rodwayi, E. rubidia, E. subcrenulata, E. urnigera, E. vernicosa* and *E. viminalis)* [35]–[37]. Although the species used in this experiment are not representative of all species within the *Symphyomyrtus* and *Eucalyptus* subgenera (which consist of over 400 and 100 species, respectively, that inhabit Australia and exhibit overlapping ranges of nutrient uptake and growth) [38], they represent a group of sympatric species that have evolved in similar environments. Further, differences in stoichiometry, physiology, and growth strategy (e.g., available foliar N, stem volume, and biomass production) between *Symphyomyrtus* and *Eucalyptus* subgenera [39]–[41] indicate phylogenetic conservatism of resource use strategy within each subgenus. Correspondingly, recent research has shown that responses to environmental change are phylogenetically conserved such that *Symphyomyrtus* species tend to gain more biomass in response to increased soil N and elevated CO_2_ than species within the subgenus *Eucalyptus* [42], [43]. *Eucalyptus nitens*, a species within the *Symphyomyrtus* subgenus that is native to mainland Australia but not to Tasmania, is gradually becoming more common in Tasmania via hardwood plantations. As shown in other commercially important tree species [44], *E. nitens* holds the potential to disperse into native stands and alter productivity and composition of native plant communities [45]. In this system and multiple others, it remains unclear how the expected doubling of global terrestrial N deposition and nutrient eutrophication in the next half century [7], [46], [47], combined with dependence of the hardwood N fertilization [48], rapidly rising atmospheric CO_2_ levels [49], and the introduction of non-native species, will interact to alter the composition and function of native plant communities.

### Experimental Design

Seed of 26 native Tasmanian eucalypt species was purchased from Forestry Tasmania (http://www.forestrytas.com.au/) (as such, no specific permits were required and no endangered or protected species were used). Seeds were vernalized by folding approximately a tablespoon of seed in a paper towel, soaking overnight in water plus a drop of dishwashing liquid (which acts as a surfactant and facilitates adhesion of water to seeds; Mason and Miller 1991), and refrigerating for 30 days at 4°C. Seeds were then sown into a commercial potting mix, with added macro- and micro-nutrients from Nutricote Grey (Langley Australia Pty Ltd., Welshpool, WA) at a concentration of approximately 3 kg/m^3^ (N∶P∶K ratio of 19∶2.6∶10) and covered with a layer of vermiculite (for water retention). After three weeks, 12 similar-sized seedlings of each species were placed into four treatments, which consisted of factorial combinations of ambient vs. elevated CO_2_ (420 ppm vs. 700 ppm) and low vs. high soil N (3 kg/ha/mo vs. 30 kg/ha/mo, applied as urea). The elevated CO_2_ and high N treatments represent levels likely to be reached by the end of the century, although increases in soil N will be spatially heterogeneous [46], [49]. We were confident that soil N concentrations in the greenhouse reflected those in the field, as plant N concentrations of species grown in a greenhouse are comparable to those of the same species grown in the field given constant fertilization regimes [50], [51]. Six of the individuals within each treatment were planted with an individual of the same species (i.e., monocultures) and the other six were planted with an *E. nitens* individual (i.e., mixtures). The elevated CO_2_ treatments were created in two greenhouse chambers: in one chamber CO_2_ was kept at an ambient level and in the other CO_2_ was elevated using compressed CO_2_ and a CO_2_ control unit (Thermoline Scientific equipment, Smithfield, Australia). To avoid greenhouse effects (pseudoreplication), the CO_2_ levels and their respective seedlings were exchanged between two chambers each week, and the CO_2_ concentrations were monitored bi-weekly with an infra-red gas analyzer (LiCor 6200, LiCor Inc., Lincoln, NE, USA). Pots were also randomly repositioned each week to avoid positional effects in the greenhouse. After six months of growth, and watering as needed, individuals from each pot were harvested and separated into aboveground and belowground biomass. Aboveground sections were weighed after 48 hours of oven-drying at 60°C, while belowground sections were weighed after careful rinsing over 2 and 0.5 mm sieves (to remove soil and retain fine root biomass) and 48 hours of oven-drying at 60°C.

### Statistical Analyses

To test our hypothesis that evolutionary history and novel biotic interactions would explain patterns in plant productivity responses to abiotic agents of global change, we analyzed whole-pot biomass (total, aboveground and belowground) and root∶shoot ratios of native species monocultures and mixtures with *E. nitens* using mixed effects models implemented in R (version 3.1.1) [52]. These models included cube root transformed biomass and root∶shoot measurements (averaged for each species within each CO_2_, N, and species pair type treatment combination) as dependent variables, and CO_2_, soil N, species pair type (i.e., native species monoculture or mixture with *E. nitens*), and native species subgenus as independent variables. Species was included as a blocking factor. Analysis of Variance (ANOVA) tables were calculated using marginal sums of squares, with significance assessed using Wald χ^2^ statistics. Because a resolved phylogenetic reconstruction of these species is not currently available [36], [37], we were unable to use phylogenetic comparative methods to address our hypothesis. Pairs in which one or both individuals died were excluded from these analyses (N = 354 total species pairs after exclusion and 190 observations after averaging across species and treatment combinations). Additionally, to quantify the magnitude of the effects of CO_2_ and soil N on productivity across native species evolutionary histories and biotic interactions, we calculated z-transformed effect sizes using native species-based differences in whole-pot total biomass between elevated and ambient CO_2_ and between high and low N. Treatment effect sizes were calculated for each subgenus (i.e., *Eucalyptus* and *Symphyomyrtus*) and species pair type (i.e., native species monocultures and mixtures with *E. nitens*).

## Results

**Figure 1:**
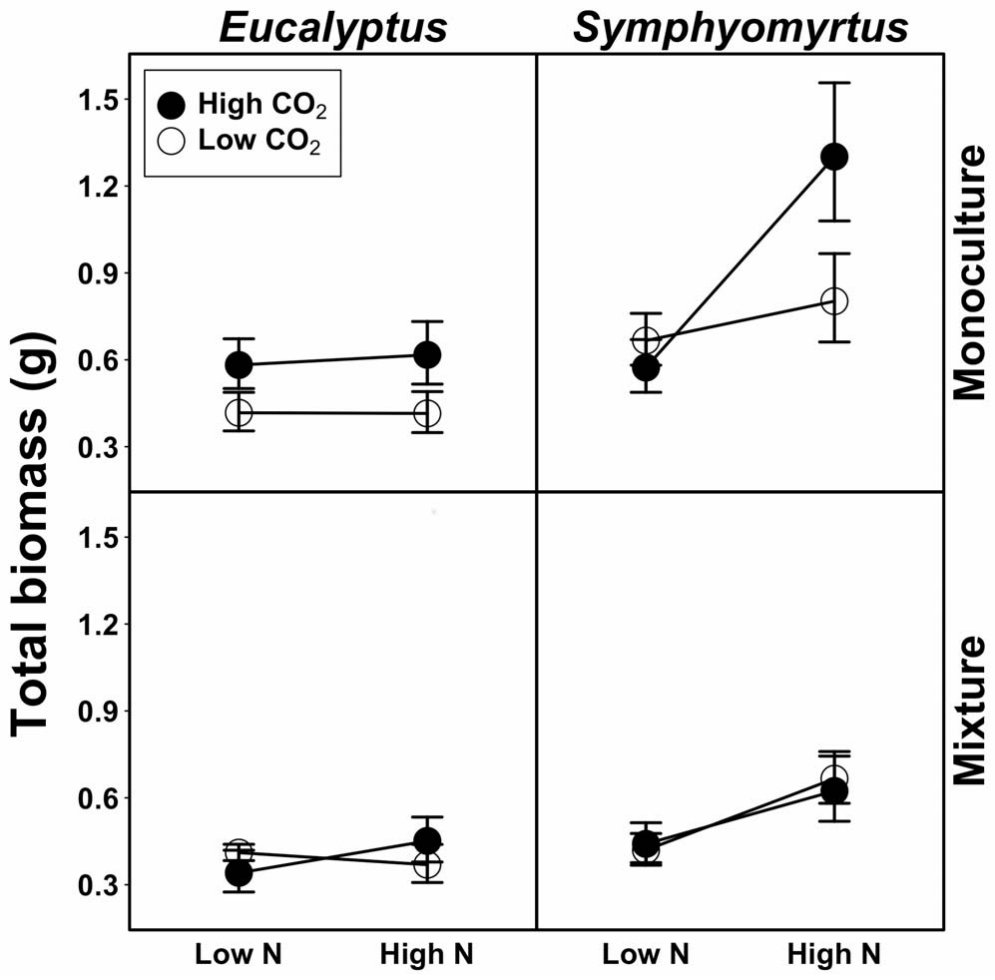
Productivity responses to global change scenarios are contingent upon species evolutionary history and novel biotic interactions. Overall, monocultures (pairs of conspecific individuals) of species in the subgenus *Symphyomyrtus* (top right panel) in elevated CO_2_ conditions exhibit the strongest responses to N. On average, these monocultures produce 126% more biomass than all other species pairs in high N and elevated CO_2_ treatments (1.301±0.205 g and 0.576±0.061 g, respectively). Above- and belowground biomass follow similar patterns. Error bars represent ±1 SEM.

Consistent with our hypothesis that plant productivity responses to abiotic agents of global change are contingent upon both evolutionary history and novel species interactions, full models of total and aboveground biomass identified a significant interaction among atmospheric CO_2_, soil N, species pair type, and subgenus (χ^2^ = 4.215_1_, p = 0.040; χ^2^ = 4.131_1_, p = 0.042) (**Table 1**; **Fig. 1**). Because of this interaction, we were unable to interpret single or two-way interactive effects identified by the models [53], [54]. Thus, we ran separate models for each subgenus that included fixed effects of atmospheric CO_2_, soil N, and species pair type, with species as a blocking factor. If we found significant interactions among factors, we ran subsequent models to better interpret the main effects (see Tables S1 and S2).

**Table 1:** Linear mixed effects model results of eucalypt productivity (total, aboveground and belowground; TB, AGB, and BGB, respectively) and biomass allocation (root to shoot ratio; R∶S) across CO_2_, soil N, species pair type (monoculture vs. mixture with the non-native *E. nitens*) treatments and native species subgenus (N = 190).

		Variable							
		δTB		AGB		BGB		R∶S	
Treatment	Df	Chisq	p	Chisq	p	Chisq	p	Chisq	p
S	1	2.63	0.105	2.458	0.117	3.42	0.064	3.78	0.052
M	1	19.995	1*10^−5^	19.428	1*10^−5^	17.966	2*10^−5^	2.851	0.091
C	1	2.191	0.139	1.78	0.182	3.518	0.061	1.789	0.181
N	1	14.749	1.2*10^−4^	15.782	7*10^−5^	7.952	**0.005**	0.141	0.707
S*M	1	1.377	0.241	1.102	0.294	1.686	0.194	0.084	0.773
S*C	1	0.205	0.65	0.151	0.698	0.494	0.482	0.358	0.55
M*C	1	5.207	**0.022**	5.722	**0.017**	3.375	0.066	0.616	0.433
S*N	1	9.377	**0.002**	10.188	**0.001**	5.427	**0.02**	0.071	0.79
M*N	1	2.4*10^−4^	0.988	0.002	0.965	9*10^−5^	0.992	0.013	0.908
C*N	1	5.513	**0.019**	5.234	**0.022**	4.803	**0.028**	0.915	0.339
S*M*C	1	0.781	0.377	0.813	0.367	0.533	0.465	0.125	0.724
S*M*N	1	0.075	0.785	0.115	0.734	0.006	0.939	0.005	0.945
S*C*N	1	0.284	0.594	0.384	0.536	0.015	0.904	0.878	0.349
M*C*N	1	0.719	0.396	0.869	0.351	0.169	0.681	0.296	0.587
S*M*C*N	1	4.215	**0.04**	4.131	**0.042**	3.364	0.067	0.785	0.376

In a greenhouse experiment, 28 native Tasmanian eucalypt species within two subgenera (S), *Symphyomyrtus* and *Eucalyptus*, were treated with factorial combinations of ambient or elevated CO_2_ (C; 420 or 700 ppm, respectively) and low or high soil N (N; 3 or 30 kg/ha/mo), and paired with a conspecific or a non-native (*E. nitens*) individual (M). In these models, whole-pot biomass measurements and ratios of root to shoot biomass were averaged for each native species in each treatment combination, cube root transformed, and blocked by species. P values are shown in bold and are significant at α≤0.05.

δ TB, total biomass; AGB, aboveground biomass; BGB, belowground biomass; R∶S, root to shoot ratio; M, species pair type (native species monoculture vs. mixture with *E. nitens*); C, CO_2_ treatment (420 or 700 ppm); N, nitrogen treatment (3 or 30 kg ha^−1^ mo^−1^).

In species pairs containing subgenus *Eucalyptus* species (N = 82), we identified a significant interaction between species pair type and CO_2_ for total and aboveground biomass (χ^2^ = 4.214_1_, p = 0.040; χ^2^ = 4.395_1_, p = 0.036) (**Table 2**). Subsequent models showed that from ambient to elevated CO_2_ conditions, monocultures (N = 45) gained 44, 40, and 68% more total, aboveground, and belowground biomass in response to elevated CO_2_ (χ^2^ = 5.437_1_, p = 0.020; χ^2^ = 6.397_1_, p = 0.011; χ^2^ = 5.153_1_, p = 0.023), whereas the biomass of mixtures (N = 37) on average did not differ between ambient and elevated CO_2_ conditions (χ^2^ = 0.134_1_, p = 0.714; χ^2^ = 0.011_1_, p = 0. 915; χ^2^ = 0. 134_1_, p = 0. 714) (**Table S1**). Moreover, we found a marginally significant interaction between CO_2_ and N in total and belowground biomass of mixtures (χ^2^ = 2.640, p = 0.104; χ^2^ = 3.607_1_, p = 0.058) (Table S1). Subsequent models showed that total and belowground biomass increased by 32 and 34% in response to soil N in elevated CO_2_ conditions (N = 18) (χ^2^ = 2.580_1_, p = 0.108), but decreased by 10 and 28% in response to soil N in ambient CO_2_ conditions (N = 19) (χ^2^ = 0.998_1_, p = 0.318) (**Table S2**; Fig. 1). These results indicate that CO_2_ stimulates growth of monocultures regardless of soil N levels, yet in mixtures CO_2_ stimulates growth when soil N is abundant and has a negative effect on growth when soil N is limiting.

**Table 2:** Linear mixed effects model results of subgenus-level eucalypt productivity (total, aboveground and belowground; TB, AGB, and BGB, respectively) and biomass allocation (root to shoot ratio; R∶S) across CO_2_, soil N, and species pair type (monoculture vs. mixture with the non-native *E. nitens*).

			Variable							
			δTB		AGB		BGB		R∶S	
	Treatment	Df	Chisq	p	Chisq	p	Chisq	p	Chisq	p
***Eucalyptus N = 82***	M	1	3.808	0.051	3.821	0.051	3.318	0.069	0.71	0.399
	C	1	2.136	0.144	1.686	0.194	3.845	**0.05**	1.762	0.184
	N	1	0.048	0.827	0.042	0.838	0.015	0.902	0.174	0.677
	M*C	1	4.214	**0.04**	4.395	**0.036**	2.897	0.089	0.337	0.561
	M*N	1	0.046	0.83	0.059	0.808	0.003	0.96	0.028	0.868
	C*N	1	1.309	0.253	1.051	0.305	1.939	0.164	1.509	0.219
	M*C*N	1	0.92	0.338	0.767	0.381	1.23	0.267	0.816	0.366
***Symphyomyrtus N = 108***	M	1	18.795	1.46*10^−5^	18.299	1.89*10^−5^	16.846	4.05*10^−5^	2.761	0.097
	C	1	0.467	0.494	0.384	0.536	0.678	0.41	0.391	0.532
	N	1	26.338	2.87*10^−7^	29.428	5.8*10^−8^	13.666	2.18*10^−4^	0.003	0.958
	M*C	1	1.414	0.234	1.653	0.198	0.841	0.359	0.162	0.688
	M*N	1	0.026	0.872	0.055	0.815	0.009	0.924	0.008	0.927
	C*N	1	4.725	**0.03**	4.941	**0.026**	2.994	0.084	0.018	0.894
	M*C*N	1	4.136	**0.042**	4.5	**0.034**	2.242	0.134	0.028	0.867

In a greenhouse experiment, 28 native Tasmanian eucalypt species within two subgenera (S), *Symphyomyrtus* and *Eucalyptus*, were treated with factorial combinations of ambient or elevated CO_2_ (C; 420 or 700 ppm, respectively) and low or high soil N (N; 3 or 30 kg/ha/mo), and paired with a conspecific or a non-native (*E. nitens*) individual (M). In these models, whole-pot biomass measurements and ratios of root to shoot biomass were averaged for each native species in each treatment combination, cube root transformed, and blocked by species. P values are shown in bold and are significant at α≤0.05.

δ TB, total biomass; AGB, aboveground biomass; BGB, belowground biomass; R∶S, root to shoot ratio; M, species pair type (native species monoculture vs. mixture with *E. nitens*); C, CO_2_ treatment (420 or 700 ppm); N, nitrogen treatment (3 or 30 kg ha^−1^ mo^−1^).

In contrast, analysis of species pairs containing subgenus *Symphyomyrtus* species (N = 108) revealed a significant interaction among CO_2_, N, and species pair type in total and aboveground biomass models (χ^2^ = 4.136_1_, p = 0.042; χ^2^ = 4.5_1_, p = 0.034) (Table 2). Subsequent models of response to CO_2_ and N for each species pair type showed a significant interaction of CO_2_ and N in total, aboveground, and belowground biomass in monocultures (N = 53) (χ^2^ = 7.273_1_, p = 0.007; χ^2^ = 4.275_1_, p = 0.039; χ^2^ = 7.821_1_, p = 0.005), but not in mixtures (N = 55) (χ^2^ = 0.017_1_, p = 0.897; χ^2^ = 0.075_1_, p = 0.785; χ^2^ = 0.006_1_, p = 0.940) (Table S1). Whereas mixtures responded positively to N in ambient and elevated CO_2_ conditions (gaining 58 and 41% more total biomass, respectively) (χ^2^ = 6.347_1_, p = 0.012; χ^2^ = 14.254_1_, p = 1.60*10^−4^), monocultures did not respond to N in ambient CO_2_ conditions (χ^2^ = 0.061_1_, p = 0.805), but gained 127% more total biomass in response to N in elevated CO_2_ conditions (χ^2^ = 32.671_1_, p = 1.09*10^−8^) (Table S2; Fig. 1). Overall, monocultures of species in the subgenus *Symphyomyrtus* receiving high N and elevated CO_2_ treatments produced 126% more biomass than all other species pairs receiving the same N and CO_2_ treatments (1.301±0.205 g and 0.576±0.061 g, respectively; Fig. 1). These results suggest that monocultures are strongly limited by N when CO_2_ levels are high, but not when CO_2_ levels are low; on the other hand, N strongly limits mixtures regardless of CO_2_ levels.

**Figure 2:**
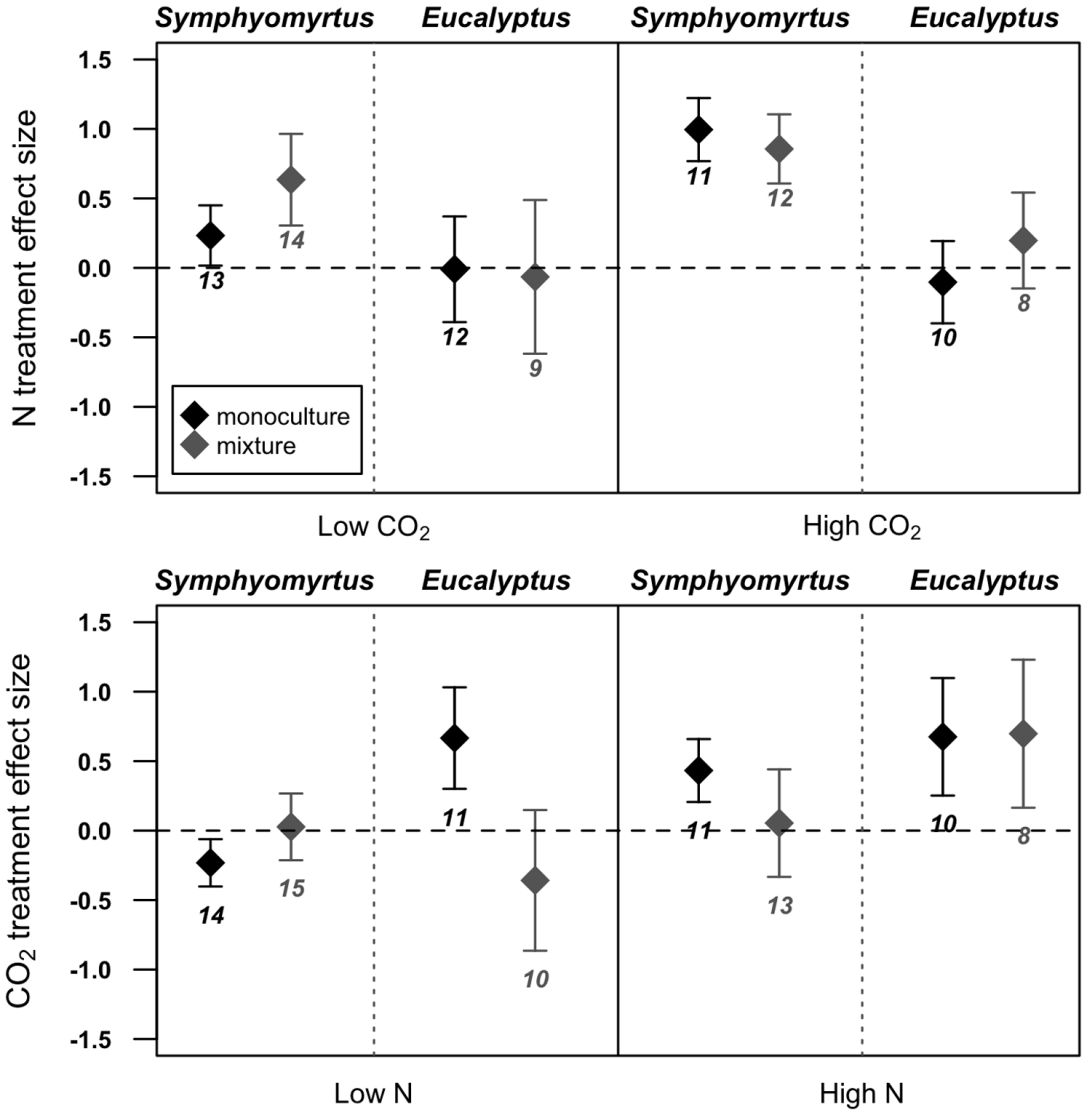
Effect sizes (standardized z-scores) of species total biomass responses to added soil N (30 kg/ha/month; upper panel) and elevated CO_2_ (700 ppm; lower panel) for native species monocultures (black) and mixtures with the non-native *E. nitens* (gray). Error bars represent ±1 SEM.

Treatment effect sizes of increased elevated CO_2_ and soil N (**Fig. 2**) broadly support results of our ANOVAs. The effect of elevated CO_2_ was positive for subgenus *Eucalyptus* monocultures in both low and high soil N levels, but was positive for subgenus *Eucalyptus* mixtures only in high soil N. Alternatively, elevated CO_2_ had a opposite effects (positive and negative, respectively) on *Symphyomyrtus* monocultures in high and low soil N conditions, and had no effect on *Symphyomyrtus* mixtures with *E. nitens*. Neither monocultures nor mixtures of species within the subgenus *Eucalyptus* responded significantly positively to increased soil N in elevated or ambient CO_2_ conditions (although mixtures exhibited slightly higher responses to N in high CO_2_ conditions), whereas those within the subgenus *Symphyomyrtus* did (although monocultures responded less positively to N in ambient compared to elevated CO_2_ conditions). These differences in response to CO_2_ and N between native species subgenera and between monocultures and mixtures with the introduced *E. nitens* support our hypothesis that both native species evolutionary history and novel interactions between native and introduced individuals shape plant responses to abiotic agents of global change.

Subgeneric differences in plant responses to increased soil N and elevated CO_2_ suggest that species within the same lineage have inherited similar traits for resource use. In support of this, we identified a marginally significant effect of subgenus on ratios root to shoot biomass in our full model (χ^2^ = 3.78_1_, p = 0.052) (Table 1). Specifically, we found that species pairs containing individuals in the *Symphyomyrtus* subgenus had an average root to shoot ratio that was 16% greater than species pairs containing individuals in the *Eucalyptus* subgenus (0.182±0.009 and 0.158±0.007, respectively). This result indicates that greater allocation towards belowground biomass in species within the *Symphyomyrtus* subgenus could be driving greater overall responses to increased soil N than species within the *Eucalyptus* subgenus; alternatively, greater allocation towards aboveground biomass in species within the *Eucalyptus* subgenus could be driving greater overall responses to elevated CO_2_ than species within the *Symphyomyrtus* subgenus.

## Discussion

The aim of this study was to address whether evolutionary history can explain responses to global change, and whether novel biotic interactions have the potential to alter evolutionarily based responses to abiotic global change factors. Drawing upon the concept of phylogenetic niche conservatism, wherein closely related species inherit the traits of their ancestors and thus occupy similar niches [20], [21], we addressed whether separate lineages respond differently to elevated atmospheric CO_2_ and increased soil N. Further, as introduced species have been shown to alter plant function in changing environments [28], [29], we examined how plant responses to elevated CO_2_ and N fertilization shift with the introduction of non-native individuals. We found that N addition increases overall productivity, especially in elevated CO_2_ conditions [1]–[4] (Table 1). However, further analysis showed that plant lineages respond differently to CO_2_ and N [8], [17], [18], [24], [42], [55] across monocultures and mixtures with an introduced species (Figs. 1 and 2). Our analysis of root∶shoot ratio across lineages indicate that this trait, and probably others important for resource acquisition and competitive ability, are evolutionarily conserved, which could explain why plant response to CO_2_ and N are contingent upon plant evolutionary background. We conclude that evolutionary history can be useful in predicting which species may be more productive with anthropogenically-driven environmental changes, but novel biotic interactions have the potential to alter these patterns. Ultimately, our results provide strong evidence that novel biotic interactions may drive unexpected patterns in carbon sequestration, and likely other critical ecosystem processes and functions, as environments change globally.

### Responses to global change through a phylogenetic lens

Consistent with previous studies, we found a strong connection between evolutionary history and response to altered abiotic conditions [14],[24],[42], suggesting that plant ecological niche space can be predicted by species phylogenetic relatedness. In their natural environments, Tasmanian eucalypt species within the *Symphyomyrtus* and *Eucalyptus* subgenera tend to co-dominate natural eucalypt stands [41], niche partitioning is a likely mechanism that has driven observed phylogenetic effects on their productivity in global change scenarios. Specifically, competitive interactions among species in the same subgenus and facilitative interactions among species in separate subgenera could have maintained unique traits such that co-occurring species would not compete for the same resources [55]. We have found that allocation of biomass above- versus belowground is an evolutionarily conserved trait that could be a key indicator of niche partitioning and response to above- and belowground environmental change. Overall, our work emphasizes that plant species responses to global change are not idiosyncratic but largely contingent upon phylogenetic relatedness; moreover, phylogenetic patterns in a group of plant species can be combined with knowledge about the ecology of those species to develop and test hypotheses about past processes (i.e., niche partitioning) that have driven their evolution.

Given that the evolution of traits involved in resource use are shaped by tradeoffs between competitive and conservative growth strategies [56], greater belowground allocation in *Symphyomyrtus* species may be accompanied by a suite of traits (e.g., lower xylem density for increased water conductance and greater specific leaf area for increased CO_2_ acquisition) that increase acquisition and rates of resource use. Ultimately, species with competitive growth strategies (in other words, greater ability to compete for and acquire resources, as exhibited in the *Symphyomyrtus* species) may be more favored by N addition and elevated CO_2_ than species with conservative growth strategies (greater ability to persist in low resource environments) as global change continues. This would alter not only community composition and diversity, but also important ecosystem processes such as nutrient cycling [57]. Thus, information about the plant species evolutionary relatedness and functional traits can potentially be useful in refining our current understanding of how plant diversity and ecosystem processes might be altered by future global change.

### Novel biotic interactions as a critical agent of global change

Human-assisted species introductions will continue to present novel plant interactions in terrestrial communities [31], [58] and our results indicate that these interactions can alter native community responses to atmospheric CO_2_ and soil N. Yet, the effects of novel biotic interactions may be unique to native evolutionary lineages. Our analysis reveals that mixtures between eucalypt species that are native to Tasmania and the introduced *E. nitens* exhibit differing responses to elevated CO_2_ and added soil N depending on the subgeneric identity of the native species (Fig. 2). Thus, the establishment of *E. nitens* individuals in native eucalypt forests may reflect a combination of abiotic agents of global change [59] as well as biotic interactions with already established species. Further, our results parallel previous studies that, in certain global change scenarios, ecological consequences are more negative for novel biotic interactions between distant relatives than those between close relatives [30], [31]. For example, in low soil N conditions, mixtures between native species within the subgenus *Eucalyptus* species (to which *E. nitens*, a symphyomyrt, is less closely related) and *E. nitens* respond less positively to elevated CO_2_ than subgenus *Eucalyptus* monocultures, whereas mixtures between native species within the subgenus *Symphyomyrtus* species and *E. nitens* respond more positively to elevated CO_2_ than subgenus *Symphyomyrtus* monocultures (Fig. 2). However, our results reveal that this pattern does not always hold across all global change scenarios. For example, in high soil N conditions, mixtures between native species within the subgenus *Eucalyptus* species and *E. nitens* respond no differently to elevated CO_2_ than subgenus *Eucalyptus* monocultures, whereas mixtures between native species within the subgenus *Symphyomyrtus* species and *E. nitens* respond less positively to elevated CO_2_ than subgenus *Symphyomyrtus* monocultures. Given these observed effects of increased soil N and elevated CO_2_ on different species combinations, current predictions of how community and global productivity will be altered in the future may be too simplistic. Thus, it is critical that both plant evolutionary history and opportunities for introduced species establishment be considered as important drivers of future carbon sequestration and multiple other important ecosystem functions [5], [7], [8], [12], [55].

The ability of introduced species to compete for resources under environmental change can determine their success in novel environments, illustrating how plasticity play an important role in introduced plant response to escalating global change [60]. As one of the most widely planted tree species in the hardwood industry, non-native *E. nitens* are high-yielding, fast growing and phenotypically adaptable to a range of environments [48]. Our results support evidence suggesting that introduced plant species have the potential to grow aggressively in novel soils [61], [62] and atmospheres, such that dispersal of *E. nitens* into native eucalypt forests may facilitate carbon sequestration as levels of soil N and atmospheric CO_2_ increase (Fig. 2). However, the ability of species to capitalize on resources may vary locally and is highly contingent upon abiotic and biotic components of destination environments [63], [64]. Though our study advances current knowledge about effects of non-native species introductions on community productivity in different global change scenarios, further research might focus on landscape-level variation in the interactions among biotic and abiotic forms of global change and the mechanisms that drive this variation. Ultimately, mosaics of past evolutionary history and contemporary abiotic and biotic interactions are fundamental to how we understand productivity across species and communities with escalating global change.

### Conclusions

The earth is experiencing an increasingly dynamic interplay of atmospheric, edaphic, and biotic changes that are already influencing plant species performance and patterns of biodiversity. Although recent research has shown that plant species evolutionary history can predict responses to one or two global change factors, outcomes of synergistically acting agents of global change are not well understood. We show that species phylogenetic relatedness and responses to multiple global change drivers can be strongly interconnected, indicating that functional traits and nutrient uptake strategies are unique to phylogenetic groups. However, given that plant communities differ in species composition and the magnitude of global change may differ across communities, collaboration among evolutionary biologists, global change biologists, ecologists and agricultural managers is critical to understand large-scale patterns in plant community responses to global change. We posit that conclusions about the future of biodiversity, composition, and function of plant communities in a rapidly changing world could be misleading if phylogenetic information and multiple agents of global change are not accounted for.

## Supporting Information

Table S1 **Models of biomass (total, aboveground and belowground) and ratio of root to shoot biomass in changing to abiotic (atmospheric CO_2_ and soil N) conditions show that the evolutionary history of native species and novel interaction with an introduced species mediate plant response to abiotic agents of global change.**
(DOCX)

Table S2 **Models of biomass (total, aboveground and belowground) and root to shoot ratio in changing soil N conditions show that species pairs respond differently to increased soil N depending on evolutionary background, interaction with an introduced species, and atmospheric CO_2_ level.**
(DOCX)
