# Supplementary material for: Evolutionary History and Novel Biotic Interactions Determine Plant Responses to Elevated CO2 and Nitrogen Fertilization
Source: PLoS One. 2014 Dec 5;9(12):e114596. doi: 10.1371/journal.pone.0114596 (PMC4257717; doi:10.1371/journal.pone.0114596)
Supplement: Table S1 — Models of biomass (total, aboveground and belowground) and ratio of root to shoot biomass in changing to abiotic (atmospheric CO2 and soil N) conditions show that the evolutionary history of native species and novel interaction with an introduced species mediate plant response to abiotic agents of global change. (DOCX) [file pone.0114596.s001.docx]

**SUPPORTING INFORMATION**

**Table S1: Models of biomass (total, aboveground and belowground) and ratio of root to shoot biomass in changing to abiotic (atmospheric CO_2_ and soil N) conditions show that the evolutionary history of native species and novel interaction with an introduced species mediate plant response to abiotic agents of global change.** In a greenhouse experiment, 28 of 30 native Tasmanian eucalypt individuals of species within two subgenera, *Symphyomyrtus* and *Eucalyptus*, were treated with factorial combinations of ambient or elevated CO_2_ and low or high soil N, and were paired with a conspecific or an *E. nitens* individual. P values are shown in bold and are significant at α ≤ 0.05.

|  |  |  |  | Variable | | | | | | | |
| --- | --- | --- | --- | --- | --- | --- | --- | --- | --- | --- | --- |
|  |  |  |  | *^δ^* TB | | AGB | | BGB | | R:S | |
| S | M | Treatment | Df | Chisq | p | Chisq | p | Chisq | p | Chisq | p |
| *Eucalyptus* | Mono (N=45) | C | 1 | 5.437 | **0.020** | 5.153 | **0.023** | 6.397 | **0.011** | 2.868 | 0.090 |
|  |  | N | 1 | 0.001 | 0.970 | 0.005 | 0.944 | 0.006 | 0.937 | 0.219 | 0.640 |
|  |  | C*N | 1 | 0.023 | 0.880 | 0.013 | 0.908 | 0.066 | 0.798 | 0.118 | 0.731 |
|  | Mix  (N=37) | C | 1 | 0.205 | 0.651 | 0.333 | 0.564 | 0.014 | 0.905 | 0.101 | 0.751 |
|  |  | N | 1 | 0.134 | 0.714 | 0.134 | 0.714 | 0.011 | 0.915 | 0.040 | 0.842 |
|  |  | C*N | 1 | 2.640 | 0.104 | 2.110 | 0.146 | 3.607 | 0.058 | 1.606 | 0.205 |
| *Symphyomyrtus* | Mono (N=53) | C | 1 | 0.701 | 0.402 | 0.768 | 0.381 | 0.545 | 0.46 | 0.086 | 0.770 |
|  |  | N | 1 | 10.787 | **0.001** | 12.589 | **3.88*10^-4^** | 4.613 | **0.032** | 0.250 | 0.617 |
|  |  | C*N | 1 | 7.273 | **0.007** | 7.821 | **0.005** | 4.275 | **0.039** | 0.013 | 0.908 |
|  | Mix  (N=55) | C | 1 | 0.059 | 0.809 | 0.139 | 0.709 | 0.009 | 0.923 | 0.122 | 0.727 |
|  |  | N | 1 | 12.049 | **0.001** | 13.007 | **3.10*10^-4^** | 7.276 | **0.007** | 0.003 | 0.955 |
|  |  | C*N | 1 | 0.017 | 0.897 | 0.006 | 0.940 | 0.075 | 0.785 | 0.038 | 0.846 |

*^δ^* TB, total biomass; AGB, aboveground biomass; BGB, belowground biomass; R:S, root to shoot ratio; S, subgenus (*Symphyomyrtus* or *Eucalyptus*); M, species pair type (native species monoculture or mixture with *E. nitens*); C, CO_2_ treatment (420 or 700 ppm); N, nitrogen treatment (3 or 30 kg ha^-1^ mo^-1^).
