# Supplementary material for: Evolutionary History and Novel Biotic Interactions Determine Plant Responses to Elevated CO2 and Nitrogen Fertilization
Source: PLoS One. 2014 Dec 5;9(12):e114596. doi: 10.1371/journal.pone.0114596 (PMC4257717; doi:10.1371/journal.pone.0114596)
Supplement: Table S2 — Models of biomass (total, aboveground and belowground) and root to shoot ratio in changing soil N conditions show that species pairs respond differently to increased soil N depending on evolutionary background, interaction with an introduced species, and atmospheric CO2 level. (DOCX) [file pone.0114596.s002.docx]

**SUPPORTING INFORMATION**

**Table S2: Models of biomass (total, aboveground and belowground) and root to shoot ratio in changing soil N conditions show that species pairs respond differently to increased soil N depending on evolutionary background, interaction with an introduced species, and atmospheric CO_2_ level.** In a greenhouse experiment, 28 of 30 native Tasmanian eucalypt individuals of species within two subgenera, *Symphyomyrtus* and *Eucalyptus* were paired with a conspecific or an *E. nitens* individual and were treated with factorial combinations of ambient or elevated CO_2_ (420 or 700 ppm) and low or high (3 or 30 kg ha^-1^ mo^-1^) soil N. P values are shown in bold and are significant at α ≤ 0.05. Similar models of species pair responses to elevated CO_2_ depending on evolutionary background, interaction with an introduced species, and soil N level showed only a significant (positive) response to elevated CO_2_ in belowground biomass of monocultures of subgenus *Eucalyptus* species (Chisq= 4.961, p=0.026).

|  |  |  |  | Variable | | | | | | | | | |  |
| --- | --- | --- | --- | --- | --- | --- | --- | --- | --- | --- | --- | --- | --- | --- |
|  |  |  |  | *^δ^* TB | | AGB | | BGB | | | R:S | | |  |
| S | M | C | Df | Chisq | p | Chisq | p | Chisq | | p | Chisq | p | |  |
| *Eucalyptus* | Mono | Low  (N=24) | 1 | 0.146 | 0.703 | 0.238 | 0.626 | | 1.00*10^-4^ | 0.991 | 0.592 | | 0.442 | |
|  |  | High  (N=21) | 1 | 0.018 | 0.892 | 0.019 | 0.891 | | 0.013 | 0.91 | 0.009 | | 0.924 | |
|  | Mix | Low  (N=19) | 1 | 1.113 | 0.291 | 0.913 | 0.339 | | 0.998 | 0.318 | 0.700 | | 0.403 | |
|  |  | High  (N=18) | 1 | 0.969 | 0.325 | 0.659 | 0.417 | | 2.58 | 0.108 | 0.916 | | 0.339 | |
| *Symphyomyrtus* | Mono | Low  (N=27) | 1 | 0.061 | 0.805 | 0.129 | 0.719 | | 0.002 | 0.961 | 0.012 | | 0.914 | |
|  |  | High  (N=26) | 1 | 32.671 | **1.09*10^-8^** | 37.785 | **7.90*10^-10^** | | 14.476 | **1.42*10^-4^** | 2.00*10^-4^ | | 0.990 | |
|  | Mix | Low  (N=29) | 1 | 14.254 | **1.60*10^-4^** | 16.311 | **5.38*10^-5^** | | 6.379 | 0.012 | 0.277 | | 0.599 | |
|  |  | High  (N=26) | 1 | 6.347 | **0.012** | 6.742 | **0.009** | | 4.215 | **0.04** | 4.00*10^-4^ | | 0.984 | |

*^δ^* TB, total biomass; AGB, aboveground biomass; BGB, belowground biomass; R:S, root to shoot ratio; S, subgenus (*Symphyomyrtus* or *Eucalyptus*); M, species pair type (native species monoculture or mixture with *E. nitens*); C, CO_2_ treatment (420 or 700 ppm).
